# Impact of an Energy Drink on the Structure of Stomach and Pancreas of Albino Rat: Can Omega-3 Provide a Protection?

**DOI:** 10.1371/journal.pone.0149191

**Published:** 2016-02-19

**Authors:** Nasra Ayuob, Rana ElBeshbeishy

**Affiliations:** 1 Anatomy Department, Faculty of Medicine, King Abdulaziz University, Jeddah, Saudi Arabia; 2 Histology Department, Faculty of Medicine, Mansoura University, Mansoura, Egypt; 3 Anatomy Department, Faculty of Medicine, Ain Shams University, Cairo, Egypt; Federal University of Rio de Janeiro, BRAZIL

## Abstract

**Background and Objectives:**

A controversy developed between the benefits of energy drinks (EDs) versus the possible health threats since its revolution. Lack of information was a call to assess the effect of chronic consumption of Power Horse (PH) as one of the EDs, on the structure of pancreas and fundic mucosa of stomach in rats, and possible protective role of Omega-3.

**Materials and Methods:**

Thirty two adult male albino rats were divided equally into 4 groups; control received group which only received a standard diet, Omega-3 group, PH group which given PH and PH plus Omega-3 group received both PH plus Omega-3 for 4 weeks. Biochemical assessment of blood glucose, serum insulin, gastrin, tumor necrosis factor alpha (TNF-α) and inducible nitric oxide synthetase (iNOS) was performed. The antioxidant activity and histopathological examination of both pancreatic tissue and fundic mucosa of stomach were assessed.

**Results:**

Administration of PH significantly increased serum insulin and glucose levels while it significantly reduced serum gastrin level compared to control. PH also caused oxidants/antioxidants imbalance in both pancreas and fundic mucosa. The latter revealed degenerative changes and increased apoptosis which was evident by increased caspase-3 immunoexpression. Pancreas exhibited signs of β-cells overstimulation. Fundic mucosa showed reduced number of parietal cells, gastrin hormone expression compared to control group. Omega-3 administration could alleviate, to some extent, these changes. It significantly decreased TNF-α, iNOS and reduced glutathione (GSH) as well as significantly increasing superoxide dismutase (SOD) and glutathione peroxidase (GPx) activities compared to the group which received PH alone.

**Conclusion:**

Power Horse intake significantly injures islet cells, pancreatic acini as well as the glandular cells of the fundic mucosa. Omega-3 decreases these detrimental effects mostly through its antioxidant and anti-inflammatory action.

## Introduction

The revolution of energy drinks (EDs) has pointed out both their popularity and controversy, given on one hand their advertized benefits of increased alertness and energy, versus their possibly crucial health threats [1–5]. Energy drinks are a group of beverages that has gained their fame since 1997 [6]. They are designed to provide the consumer by a combination of stimulants and energy boosters that increases the physical endurance, concentration and sustenance; improves cognitive as well as muscular performance and provide mood enhancement [7, 8]. Insufficient sleep (67%) and the desire to increase energy (65%) were the most common reasons for their consumption [2].

Energy drinks mostly contain caffeine, other plant based stimulants (guarana, ephedrine, yerba mate), sugars and their derivatives (glucose, fructose, sucrose, ribose and glucuronolactone; which is a naturally occurring glucose metabolite), amino acids (taurine, carnitine, creatine), other herbal extracts (ginseng, ginkgo biloba), maltodextrin, inositol, vitamin B complex and other ingredients [6, 9]. Due to the vast array of ingredients forming EDs, their side effects are expected to be much more than beverages that contain caffeine alone [10]. Caffeine, one of the most commonly worldwide consumed alkaloids present in coffee, tea or soft drinks [11], that causes gastrointestinal upset such as heart burn, increased esophageal reflux and gastric secretion with susceptibility to ulceration, both in acute and chronic intoxication [9,12]. In addition to other stimulants as taurine, a sulphur-containing amino acid found in most mammalian tissues that enhances the effects of caffeine [13, 14]. Also, the high sugar content that forms 10–13% of the volume of EDs leads to obesity and diabetes [9, 13]. Young adults and adolescents are particularly attracted to EDs, influenced by the marketing with lack of knowledge of the potential risks [2, 15]. There is little published literature on the adverse effects of ED and they were recently given unique reporting codes, so their toxicity can be tracked [16]. Germany has tracked EDs—related incidents since 2002 and many harmful outcomes have been reported [17].

Omega-3 in Fish oil is one of the most important polyunsaturated fatty acids (PUFA) that have an anti-inflammatory and an antioxidant activity [18, 19]. It is a blend of two essential fatty acids: eicosapentaenoic acid (EPA) and docosahexaenoic acid (DHA) [20], that has essential role in maintaining good health and in decreasing chronic inflammatory diseases such as inflammatory bowel disease and various inflammatory gastrointestinal diseases [21–24]. Moreover, as one of the essential fatty acids, Omega 3 contributes in the protection of the gastroduodenal mucosa [25]. On the other hand, Omega-3 PUFAs are prime candidates for environmental modulators of type I diabetes [26]. Also, recent studies suggested that dietary intake of Omega-3 could be useful in prevention of diabetes; as it reduced the activity of the pro-inflammatory processes which stimulated the body to attack its own insulin-producing cells [27, 28]; hence it was used in this study.

With EDs becoming a worldwide phenomenon, the short- and long-term effects of these beverages must be evaluated more closely in order to fully comprehend their impact on different body organs. Adding to that, the acute and chronic effects resulting from the prolonged intake of their additives are not well identified. Therefore, the aim of this study was to assess the impact of Power Horse (PH), as one of the commonly used EDs, on the pancreas and fundic mucosa of stomach in albino rats, elicit the possible mechanism and to determine the possibility of a protective role for Omega-3.

## Materials and Methods

### Chemicals

Power Horse (PH); one of the commonly used EDs available in the Saudi Market, was used in this study in a dose 10mg/kg according to Akande and Banjoko [29]. This dose for rats was equivalent to the human dose according to Paget and Barnes [30] conversion tables. The (PH) contains caffeine, taurine, glucuronolactone, sugars and sweeteners, color (caramel) flavoring, vitamins, inositol, niacin, herbal supplements, and other ingredients [3, 31]. Omega-3 fish oil capsules, purchased from Wassen international Ltd Company UK, were used in this study. Each capsule includes 350 mg fish oil of which 100 mg omega-3 fatty acids, 49 mg elcosapentaenoic acid (EPA), 35 mg docosahexaenoic acid (DHA). These capsules were used to avoid many variables that can arise from diet and feeding procedures, including impurities in the oils used, food storage, and diet duration.

### Animals

This experimental study was approved by the research ethics committee at King Fahd Medical Research Center (KFMRC), King Abdulaziz University, Jeddah, Saudi Arabia. Thirty two adult male Wistar albino rats, with a body weight ranged from 230 ± 20 g supplied from KFMRC were used in this study. All animals were housed in suitable plastic cages, at a controlled temperature (24±1°C), 70% relative humidity and air flow conditions with fixed 12 hour light-dark cycles for one week before the experiment for acclimatization on the laboratory conditions. Fresh water *ad libitum* and standard rodent food pellets were always available. The animals were divided into 4 groups (n = 8 each). The control group (GIa) received 7.5 ml saline using a gastric tube once daily for 4 weeks. The Omega-3 treated group (GIb) was used as positive control and was given fish oil by a gastric tube, at a dose of 300 mg/kg (equivalent to 0.05–0.04 ml fish oil/rat once daily for 4 weeks) [32]. The PH treated group (GIIa) was administrated 10mg/kg PH equivalent to 7.5 ml once daily for 4 weeks using a gastric tube [29]. The PH plus Omega-3 treated group (GIIb) received similar daily doses of both PH and Omega-3 for 4 weeks.

All rats were weighed at the end of each week. During the last day of the experiment, animals were deprived of food overnight then anaesthetized by mild ether inhalation and subsequently sacrificed by cervical dislocation. Blood samples were taken directly from the heart for biochemical assessment. The abdomen of the rats was opened, the pancreas was dissected out, the stomach was perfused with cold saline, tied at the esophageal and duodenal junctions, cut at the two ends and put intact in a deep petri-dish. The stomach was opened through the greater curvature, rinsed in two changes of ice-cold normal saline, cut into longitudinal strips and fixed together with the pancreas in 10% neutral buffered formalin overnight then processed to obtain paraffin blocks. Parts of both organs were kept at -80°C for estimation of biochemical markers. Serial paraffin sections, at 3–4 μm thickness, were obtained from the paraffin blocks and stained with hematoxylin and eosin (H&E) for the histopathological examination [33].

### Biochemical analysis

Blood samples were left undisturbed for 30 min then centrifuged at 4000 rpm for 15 min at room temperature. Serum was collected and kept at −80°C until time for assay. Serum glucose was determined using the hexokinase method and insulin was determined by means of an enzyme-linked immune-sorbent assay. Serum gastrin levels were determined using a competitive immunoassay technique using a DRG rat gastrin kit (DRG International, Inc., New Jersey, USA). Calculating the homeostasis model assessment of insulin resistance (HOMA-IR), was done based on the formula; HOMA-IR = serum glucose (mg/dL) × plasma insulin (μU/mL)/405 [34].

The serum TNF-α and inducible nitric oxide synthetase (iNOS) levels were measured using ELISA kits (R&D Systems) according to the manufacturer's instructions. Pancreatic tissue and fundic mucosa homogenate was prepared for assessment of iNOS concentration, using assay kits (Nanjing Jiancheng Bioengineering Institute) according to manufacturer’s instructions. In addition, the levels of reduced glutathione (GSH), glutathione peroxidase enzyme activity (GPx) and superoxide dismutase enzyme activity (SOD) were assessed in the tissue homogenate using Biodiagnostic kits, Egypt according to the manufacturer's instructions.

### Immunohistochemistry (IHC) assessment

Immunohistochemical studies were carried out using the peroxidase-labeled Streptavidin—Biotin Technique according to Ramos-Vara et al. [35]. Paraffin sections were deparffinized and rehydrated down to distilled water then they were treated in 3% Hydrogen peroxide (H_2_O_2_) for 5 min and rinsed with phosphate buffer solution (PBS) for 15 min. The sections were blocked with 1.5% normal goat serum in PBS then incubated for 45 min at room temperature with the primary antibody. Anti-caspase-3 mouse monoclonal antibody (Dako Company, Cairo, Egypt, Catalog No. IMG-144A at a dilution 1/200) was used for detection of apoptosis (pro and active). The anti-gastrin antibody (A 0568 Dako Cytomation Denmark) was used at a dilution (1:800). The anti-insulin monoclonal mouse primary antibody (DAKO LSAB 2 Kit; Dako, Denmark) at a dilution of 1:100 was also used. Sections were subsequently incubated with a second-stage biotinylated antibody (biotin-conjugated goat anti-rabbit IgG, at a dilution of 1:200) for 1 h, at room temperature. After rinsing in PBS, the reaction products were visualized by immersing the section into the chromogen diaminobenzidine. Finally, the sections were counterstained with hematoxylin, dehydrated and covered. Slides stained with secondary antibody IgG only were used as negative controls.

### Morphometric and statistical analysis

An Olympus Microscope BX-51 with a digital camera connected to a computer with an image analyzer system software (Pro Plus image analysis software version 6.0) was used for photographing and morphometric study in the microscope center, KFMRC. In the pancreas, the areas of 10 non-overlapping islets were measured in three serial H&E-stained sections at a magnification ×100 as described by [36]. In each group, the mean area percent (AP) and mean intensity (MI) of insulin immunoexpression of at least 20 islets per animal were measured and analyzed at a magnification x100 [37]. At the same magnification, the AP and MI of caspase-3 immunoexpression were assessed in 5 non-overlapping pancreatic sections examined for each rat in each group and 10 readings were calculated [38].

Similarly, 10 readings from 5 non-overlapping H&E-stained sections, taken from each rat of each group, were measured at a magnification of x200, to count the parietal cell number in the fundic glands of the stomach. Using the same magnification, the gastric mucosal height (the perpendicular distance between the gastric mucosal surface and the muscularis mucosa) was measured in 5 fields observed for each rat; subsequently 20 measurements were calculated [39]. The AP and MI of caspase-3 and gastrin immunoexpression were assessed at same magnification (x200) and 10 readings were recorded from 5 non-overlapping gastric sections examined for each rat.

### Statistical analysis

Statistical analysis was performed using SPSS software, version 16.00 (Chicago, Illinois, USA) (S1 File). All data were expressed as mean±SD. One-way analysis of variance (ANOVA) and post-hoc with least significant difference were used for comparison between groups. Significance was considered at p<0.05.

## Results

### Effect of PH on body weight

**Fig 1:**
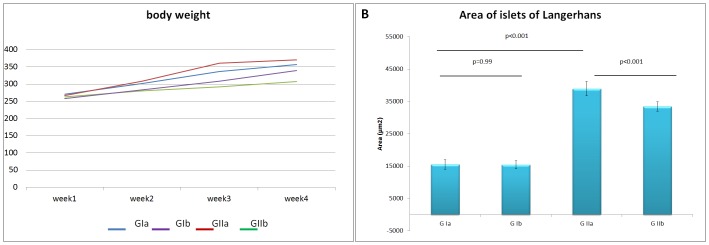
A. Mean body weights of the studied groups during the experiment. B. Mean area of islets of Langerhans. Note: There is a significant increase in GIIa compared to GIa and a significant decrease in GIIb compared to GIa. (GIa; control, GIb; receive Omega-3, GIIa; received PH and GIIb; received PH plus Omega-3).

There was no significant change in the mean body weights of rats of all studied groups during and at the end of the 4 weeks of PH administration (Fig 1).

### Effect of PH on serum insulin, glucose and gastrin levels

A significant increase (p<0.001) was observed in the serum insulin and blood glucose levels in rats which were given PH compared to the control. On the other hands, both parameters were significantly decreased (p = 0.03, p = 0.01) in rats which received PH plus Omega-3 compared to those that were given pH alone. Rats which were given PH presented a significantly higher (p<0.001) HOMA-IR index compared to their control rats. This increase was prevented in the rats received PH plus Omega-3 (Table 1).

**Table 1:** Serum insulin, gastrin, blood glucose, iNOS, TNF-α and levels of the studied groups at the end of the experiment.

Parameters	Control group (GIa)	Omyga-3 group(GIb)	PH group (GIIa)	PH plus Omyga-3 group (GIIb)
**Insulin (μIU/ml)**	5.07±0.5	4.91±0.12	17.7±1.15^b^	15.47±0.99^c^
**Glucose (mg/dl)**	113.81±6.34	112.3±1.94	167.19±10.43^b^	152.8±11.6^c^
HOMA-IR*	1.42±0.15	1.36±0.04	7.22±0.75^b^	5.84±0.62^c^
**Gastrin (Pg/ml)**	97.6±3	99.7±4.3	63.1±2.5^b^	69.8±6.6^c^
**iNOS (pg/ml)**	1.2±0.27	1.11±0.13	4.5±.8^b^	2.4±0.7^c^
**TNF-α (pg/ml)**	309.5±11.5	285.1±42.6	430±61.1^b^	330.9±60.7^c^

Results were expressed in the form of Mean±SD

^b^, significant change in GIIa compared to GIa.

^c^, significant change in GIIb group compared to GIIa.

Significance considered at (p<0.05)

iNOS: inducible nitric oxide synthetase.

TNF-α: Tumor necrosis factor alpha

PH: Power Horse

* HOMA-IR = Glucose (mg/dl) x Insulin/405.

When it came to serum gastrin, there was a significant decrease (p<0.001) observed in the rats which were administrated by PH compared to the control while it was significantly increased (p = 0.02) in rats that were given PH plus Omega-3 compared to those received pH alone (Table 1).

### Effect of PH on serum iNOS and TNF-α level

Administration of PH resulted in significant increase in the levels of iNOS and TNF-α level compared to the control. On the other hand Omega-3 administration along with PH resulted in significant reduction in their levels compared to the group received PH alone (Table 1).

### Effect of PH on antioxidant activity in pancreas and fundic mucosa

A significant increase in GSH and iNOS levels as well as a significant decrease in SOD and GPX in pancreatic tissue and fundic mucosa was observed following administration of PH for 4 weeks. Simultaneous administration of PH plus Omega-3 resulted in a significantly lower levels of GSH and iNOS as well as significantly higher levels of SOD and GPX compared to those which were given PH alone (Table 2).

**Table 2:** Reduced glutathione (GSH), superoxide dismutase (SOD), glutathione peroxidase (GPX) and induced nitric oxide synthetase (iNOS) levels in pancreas and fundic mucosa.

Parameters	Controlgroup (GIa)	Omyga-3group (GIb)	PH group (GIIa)	PH plusOmyga-3group (GIIb)
**Pancreatic GSH (U/g tissue)**	0.46±0.15	0.39±0.08	0.63±0.11^b^	0.48±0.14^c^
**Pancreatic SOD (U/mg tissue)**	84.6±10.7	91.4±19.5	52.2±16.1^b^	77.8±11.1^c^
**Pancreatic GPX (U/g tissue)**	0.49±0.15	0.54±0.15	0.33±0.07 ^b^	0.44±0.11.3^c^
**Pancreatic iNos (U/mg tissue)**	0.53±0.16	0.49±0.14	1.22±0.28^b^	0.75±0.12^c^
**Fundic mucosa GSH (U/g tissue)**	24.8±9	23.1±8.6	49.1±13.6^b^	31.9±7.3^c^
**Fundic mucosa SOD (U/mg tissue)**	93.8±20.1	102.6±18.9	62.3±20.5^b^	89.3±14.1^c^
**Fundic mucosa GPX (U/g tissue)**	0.19±0.07	0.21±0.06	0.08± 0.05^b^	0.12±0.04^c^
**Fundic mucosa iNos (U/mg tissue)**	0.42±0.13	0.36±0.11	1.32±0.23^b^	0.87±0.12^c^

Results were expressed in the form of Mean±SD

^b^, significant change in GIIa compared to GIa.

^c^, significant change in GIIb group compared to GIIa.

Significance considered at (p<0.05)

PH: Power Horse

### Effect of PH on the histologic structure of the pancreas

**Fig 2:**
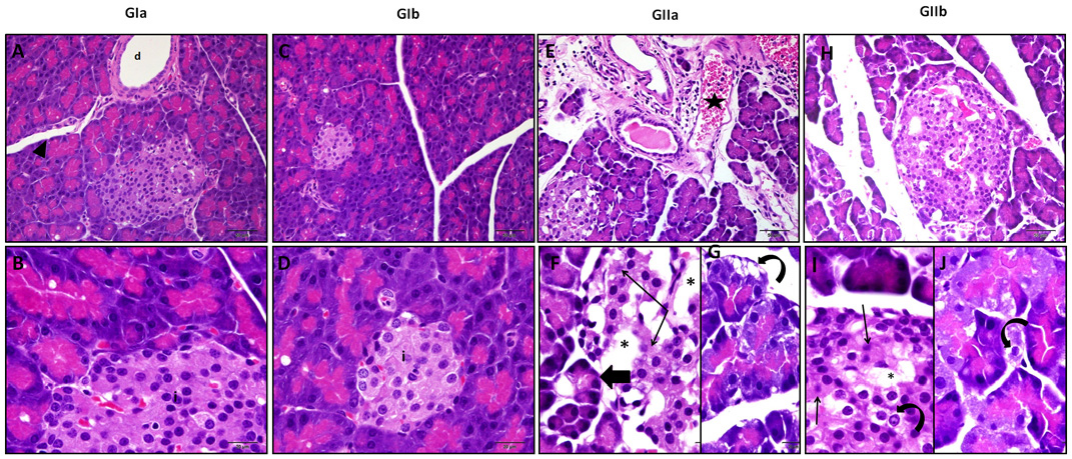
Pancreas of control (A, B) and Omega-3 (C, D) groups show intact histologic structure. Pancreas of the group which received PH (E, F, G) reveals dilated blood vessels (star) with perivascular inflammatory cell infiltrate. The islets of Langerhans exhibit empty spaces (asterisk) and some cells with lost or karyolitic nuclei (thin arrow). Some pancreatic acini appear small and atrophied (thick arrow) with dark nuclei whereas others show vacuolation (curved arrow). The islets of group received PH plus Omega-3 (H, I, J) display some vacuoles (asterisk), vacuolated cells (curved arrow) and cells with karyolitic nuclei (thin arrow) (H&E A,C,E,H X200,B,D,F, G,I,J X1000). (GIa; control, GIb; receive Omega-3, GIIa; received PH and GIIb; received PH plus Omega-3).

Omega-3 administration has no effect on the histological structure of the pancreas compared to that of the control (Fig 2).

**Fig 3:**
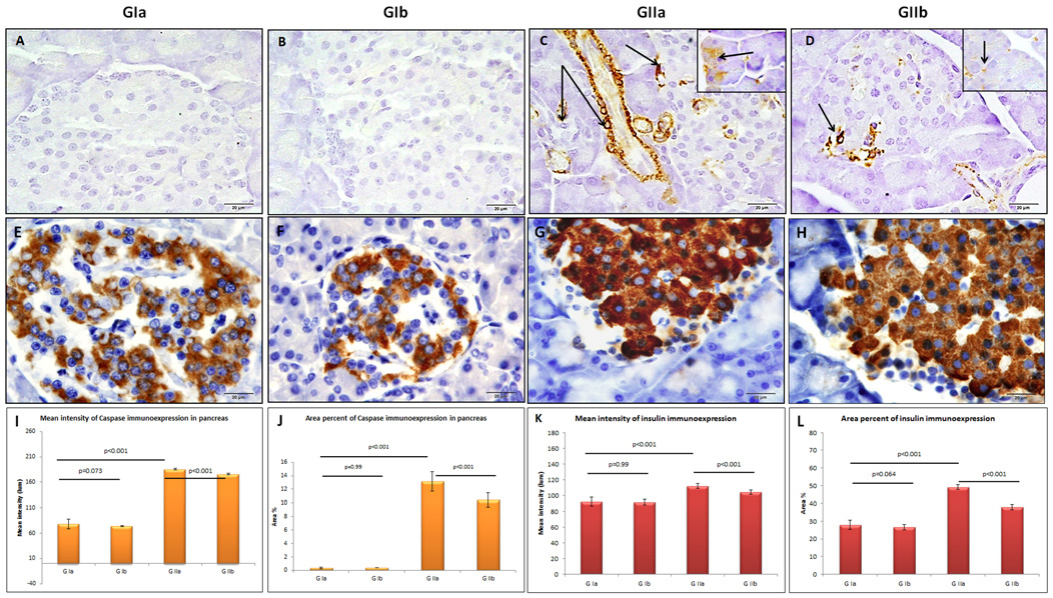
Pancreas of control (A) and Omega-3-treated (B) groups show very few caspase-3 immuno-positive reaction in both acinar and islet cells whereas that of PH group (C) reveals a strong cytoplasmic caspase-3 reaction (arrows) in some ductal, acinar and islet cells. Fewer number of caspase-3 positive cells (arrow) are noticed in islets and acini of group received PH plus Omega-3 (D). The insert displays caspase-3 reaction in the acini (Caspase-3 immunoreactivity X 1000). Islets of Langerhans of control (E) and Omega-3-treated (F) groups show moderate insulin immune-expression in β cells while those of PH group which received PH alone (G) or plus Omega-3 (H) exhibit a strong positive reaction (Insulin immunoreactivity × 1000). Histograms present mean intensity (I) and area percent (J) of caspase-3 reaction in the pancreas as well as mean intensity (K) and area percent (L) of insulin expression in the islets of Langerhans. (GIa; control, GIb; receive Omega-3, GIIa; received PH and GIIb; received PH plus Omega-3).

Pancreatic islets of Langerhans of rats which were given PH showed marked necrotic changes and vacuoles. Karyolysis, which means disappearance of nucleus, was observed. A significant increase in the area of islets was recorded in this group compared to the control (Fig 1). The dilatation and congestion of blood vessels with perivascular inflammatory cell infiltrate were obvious. The pancreatic acini appeared small with dark nuclei, vacuolated cells, lost apical acidophilia which mostly resulted from decreased zymogen granules (Fig 2). A strong positive caspase-3 immuno-expression was observed in the cytoplasm of some ductal, acinar and islets cells of this group together with a significant increase in both MI and AP of caspase-3 expression, compared to the control one (Fig 3). A strongly positive insulin immuno-expression was observed in this group whereas it was moderately positive in the control group. A statistically significant increase was recorded in both MI and AP of insulin expression in this group compared to the control (Fig 3). Similar changes but to a less extent were also detected in the pancreatic tissue of rats received PH plus Omega-3 (Figs 2 and 3).

### Effect of PH on the histologic structure of the fundic mucosa

**Fig 4:**
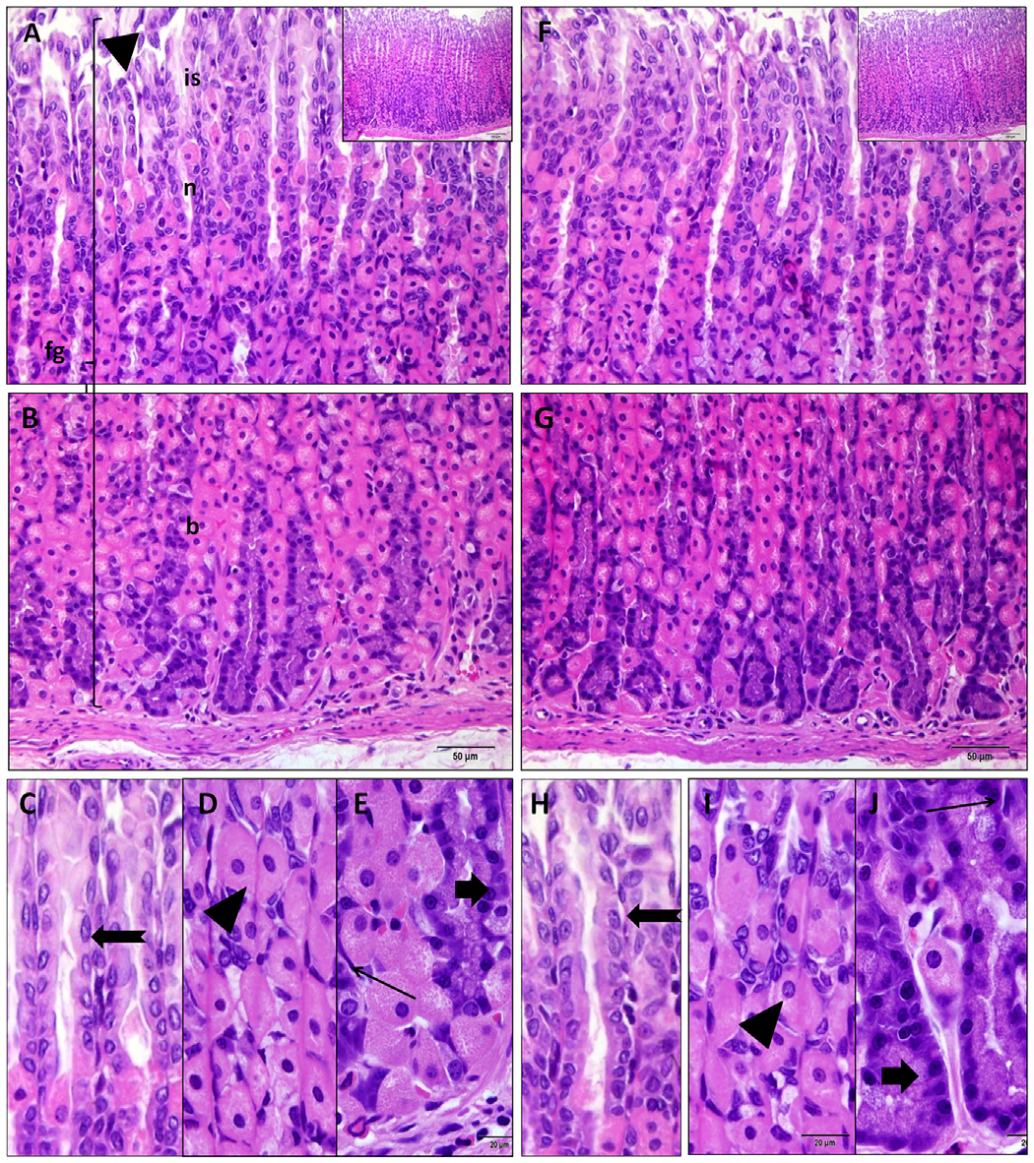
Fundus of the stomach of control (A, B) and Omega-3-treated (F-J) groups showing regularly arranged fundic glands with gastric pits (arrow head), isthmus (is), neck (n), and body (b). The upper part of the glands (C) reveals surface mucous cells (bifid arrow), the middle part (D) has many parietal cells (arrow head) and the basal part (E) shows multiple chief cells (thick arrow) and fibroblasts (thin arrow) (H&E A,B,F,G x400, C-E, H-J x1000, insert x200).

**Fig 5:**
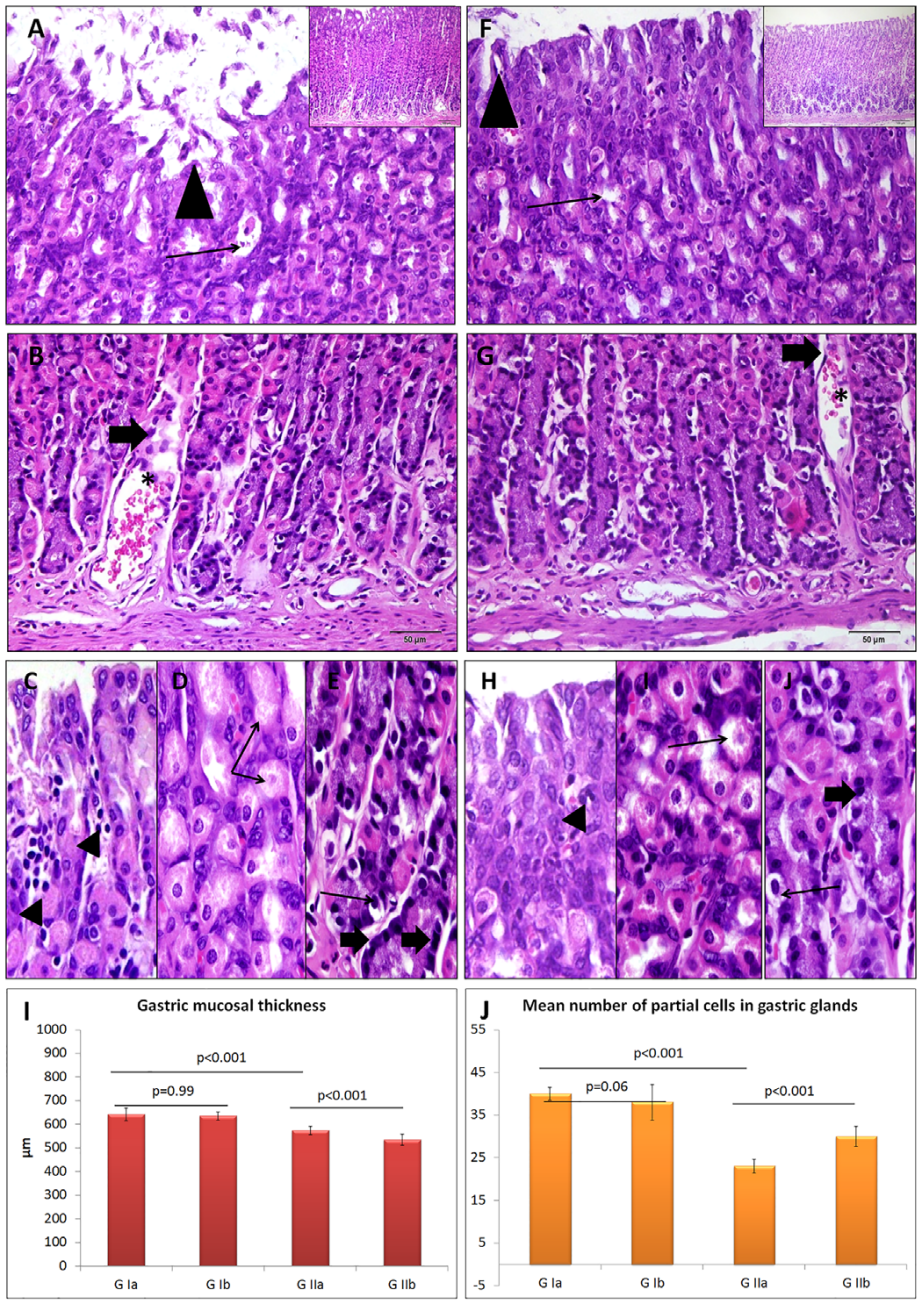
Upper part of fundic glands of the group received PH (A) exhibits minute gastric ulcer (arrow head) with desquamation of the lining epithelium into the lumen and many vacuolated glandular cells (arrow). The basal part (B) of the glands shows engorged blood capillaries (asterisk) in the lamina propria at the base of atrophied fundic gland (thick arrow). Higher magnification of the upper (C), middle (D) and basal (E) parts of the glands reveals inflammatory cell infiltrate (arrow head) between the glands, vacuolated parietal cells with lost nuclei (arrow) and dark chief cell pycknotic nuclei (thick arrow). Similar changes are observed in fundic glands of PH plus Omega-3 group but with less intensity (F-J) (H&E A,B,F,G x400, C-E, H-J x 1000, insert x200). Histograms present fundic mucosal thickness (I) and mean number of parietal cells (J) in the studied groups.

**Fig 6:**
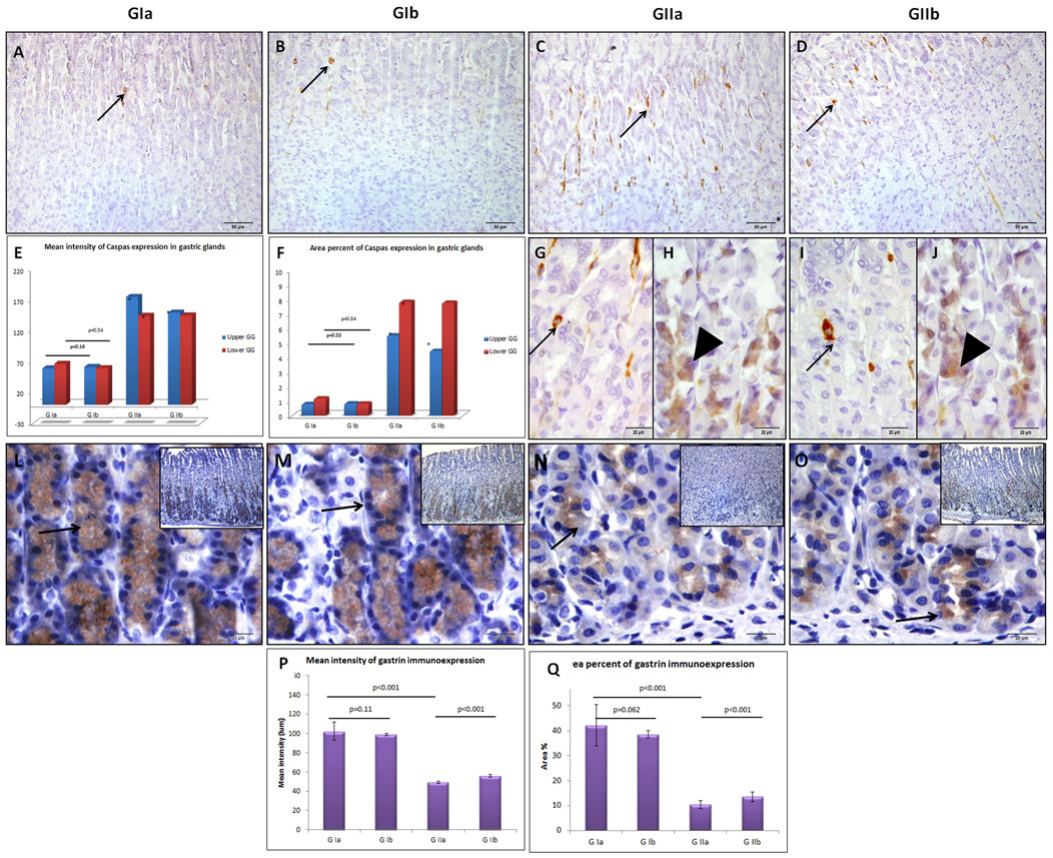
Fundic glands of control (A) and Omega-3-treated (B) groups display caspase-3 immune-positive reaction (arrows) in some glandular cells mostly in upper part of the gland, while those of the groups which received PH alone (C) or plus Omega-3 (D) show positive reaction (arrows) in many glandular cells in upper and middle parts the glands. Higher magnifications of the same groups reveal strong reaction (arrows) in many glandular cells in the upper part (G,H) and a moderate reaction in the middle parts (I,J) of the glands (arrow head) (Caspase-3 immunoexpression A-D ×400, G-J x1000). Histograms present mean intensity (I) and area percent (J) of caspase-3 reaction in the fundic glands. Fundic glands of control (L) and Omega-3-treated (M) groups exhibit strong positive gastrin expression (arrows) in the cytoplasm of many cells in the lower part of the gland, while those of the PH group (N) reveal showing few cells with moderate to weak expression. Some glandular cells of the group which received PH plus Omega-3 (O) show moderate gastrin expression (gastrin immunoreaction x1000, insert x 200). Histograms reveal mean intensity (I) and area percent (J) of gastrin expression. (GIa; control, GIb; receive Omega-3, GIIa; received PH and GIIb; received PH plus Omega-3).

No histological changes were detected in the fundic mucosa of the Omega-3 treated group compared to the control group (Fig 4). Minute gastric ulcers with desquamation of the lining epithelium were observed in fundic mucosa of rats that received PH. The upper part of the glands exhibited inflammatory cell infiltrate while the middle and basal parts showed vacuolated parietal cells with lost nuclei as well as some dark chief cells with dark pyknotic nuclei. The lamina propria presented engorged blood capillaries and few atrophied glands. Both mucosal thickness and number of parietal cells were significantly decreased compared to the control rats (Fig 5). The fundic glands of the PH-treated group revealed positive caspase-3 immuno-expression in many glandular cells, which was more obvious in the upper and middle parts of the glands. The MI and AP of caspase-3 expression were significantly increased all over the fundic glands (both upper and lower parts) in the PH-treated group compared to the control (Fig 6). The degree of gastrin immuno-expression was weak to moderate in most of the cells in the basal parts of the fundic glands of PH-treated group, while the control showed strong positive expression in these cells (Fig 6). Both MI and AP of gastrin expression were significantly decreased in the fundic glands of the PH group compared to the control one (Fig 6). The same changes were observed in the fundic glands of rats which received PH plus Omega-3 but with less extent (Figs 5 and 6).

## Discussion

Consumption of energetic drinks, which are rich in caffeine, is increasing among young individuals. At a high concentration of caffeine (500mM) a pro-oxidant environment in Sertoli cells was induced and was accompanied by an increase in proteins oxidation [40]. In this study the effect of the ED was selectively studied on the pancreas and gastric mucosa of the rat. We hypothesized that ED, due to its high content of caffeine, induces a pro-oxidant environment. The fish oil Omega-3 was selected to study its ability to protect against this effect as it was proved to be beneficial for preventing oxidative stress-induced apoptosis of gastric epithelial cells [41] and pancreatic acinar cells [42] specifically.

In the present work, the effect of PH, one of the EDs, on the histological structure of the exocrine part of pancreas, ß cells of islets of Langerhans and fundic mucosa of stomach in adult male albino rats was assessed. The study revealed that, PH significantly increased serum insulin and glucose levels and produced signs of degeneration of variable degrees in the islets' cells and pancreatic acini as well as in the glandular cells of the fundic mucosa. It reduced the antioxidant capacity in these two organs. Omega-3 succeeded, to some extent, to ameliorate these histopathologic and biochemical changes. This study reported no significant change in the body weight of rats that received EDs for four weeks, similar to the observation of Ebuehi et al. [43] in rabbits after oral administration of EDs, including PH, for almost the same period of time. In the present study, serum insulin and glucose levels were significantly increased in the rats that received PH. Similarly, Sadowska [44] and Crişan et al. [45] reported hyperglycemia in rats which ingested EDs for 2 and 6 weeks respectively. Acting synergistically, the essential ingredients of EDs; sugar and caffeine increased the postprandial hyperglycemia [46]. It was reported that oral administration of EDs as PH and red bull to rabbits might alter cholinergic neurotransmission and neural functions mediated by acetylcholine thus increasing glucose concentration [43]. In addition, the combination of high sugar content or carbohydrate rich diets with niacin as in EDs might affect carbohydrates metabolism and lead to diabetes outbreak [47].

Interestingly, in the current work, the glucose level was increased inspite of elevated insulin level that was verified biochemically, morphometrically and immunohistochemically. Regulation of islet cell proliferation in vivo was influenced by the relationship between insulin and glucose [47]. It seems that over stimulation of insulin-secreting cells in response to chronic ED administration was followed by increased insulin resistance, that was confirmed by the significant increase in HOMA-IR- calculated in this study, and cell exhaustion which might result in diabetes mellitus later on. This hypothesis was endorsed by Sadowska [44] who suggested that the hyperglycemia together with lower fat content in rat muscles after 6 week of ED ingestion was characteristically due to metabolic changes which enhanced lipolysis and development of insulin resistance. Insulin resistance could be triggered by intake of caffeine [48], niacin [49], both are ingredients of EDs. The action of caffeine could be through several mechanisms included; reduction of tissues’ sensitivity to insulin, impairment of glucose metabolism and stimulation of the secretion of stress hormones, as adrenaline and cortisol; that increase blood glucose level, lipolysis, glucogenesis, along with reduction of peripheral glucose consumption by inhibiting the activity of key glycolytic enzymes [44]. Under hyperglycemic conditions, glycation of phospholipids in the cell membrane or the organelles occur which causes oxidative stress (lipid peroxidation) in organs [50]. This oxidative stress condition was documented, in this study, as an increased production of GSH and iNOS and reduced production of GPX and SOD in both pancreas and fundic mucosa.

In the present work, PH intake produced signs of degeneration of variable degrees in islets' cells and many pancreatic acini as well as in the glandular cells of the fundic mucosa. Among these degenerative changes were the intracytoplasmic vacuoles that were also described in the hepatocytes [51] and peripheral blood cells of adult rats [52], following 2 and 4 weeks of PH ingestion. These intracellular vacuolization were also reported in the rat submandibular salivary glands [53], as well as in the papilla of rat kidney [54] following oral intake of ED. Similar to our findings in the pancreas and fundic mucosa, leucocytic infiltration and congestion of blood sinusoids were observed by Khayyat et al. [52] in the liver of rats consuming EDs including PH for 4 weeks. They referred them to the interaction that might occur between diverse constituents of EDs.

The gastric mucosal desquamation, shedding, minute ulcers, atrophic glands and congestion of blood vessels observed in the ED treated group of the current work might clarify the harmful effect of the PH on the gastric surface epithelium which forms a physical barrier between the lumen and underlying mucosa. Although no previous studies were found to investigate the structural effect of EDs or its constituents on the gastric mucosa, it is worth mentioning that in a five year period between February 2005 to December 2009, the national New Zealand poison center received 20 out of 82 calls (approximately a quarter) relating the consumption of EDs to nausea, vomiting and abdominal pain [55]. Stomach upset and diarrhea are common forms of gastrointestinal tract irritation resulting from caffeine ingestion [56]. The inhibitory effect of caffeine on gastric mucosal mucus secretion may be one of the important factors of the gastric mucosal injury, in addition to the known stimulating effect of caffeine on gastric acid secretion [57], the increased acidity exerts a negative feedback mechanism which inhibits the gastrin release [58]. This was amplified in this work as the gastrin hormone level was significantly decreased biochemically and immunohistochemically in PH treated group. Despite the high content of caffeine in EDs, which amounts approximately 3 times that in cola drinks per servings, EDs often contain additional amounts of caffeine through additives, including guarana, kola nut, yerba mate, and cocoa. Manufacturers are not required to list the caffeine content from these ingredients [59]. Thus, the actual caffeine dose in a single serving may exceed that listed [2].

It was observed, in this study, that Omega-3 succeeded, to some extent, to protect the pancreas and fundic mucosa from the deteriolatinf effects of the PH-induced biochemical and histopathological changes. Omega-3 could specifically reduce the number (indicated by the area percent) and intensity (indicated by the mean intensity) of caspase-3 immunoexpression denoting apoptotic cellular rescue in both studied organs. These findings are in line with the previous two studies which declared that Omega-3 fatty acids were beneficial for preventing oxidative stress-induced apoptosis by inhibiting apoptotic gene expression and DNA fragmentation of gastric epithelial cells [60] and pancreatic acinar cells [61]. The anti-inflammatory activity of Omega-3, evident by significantly reduction in the pro-inflammatory mediators, iNOS and TNF-α was observed in this study. Suresh and Das reported that Omega-3 PUFA may lower inflammation susceptibility and dampen the inflammatory response in the pancreatic tissue by suppressing cytokine production [42]. Adding to that the anti-inflammatory effects of Omega-3 that were previously reported by Calder [21] and Wall et al. [62]. In addition, Omega-3 antioxidant activity, evident by significant reduction in GSH and iNOS levels as well as significant increase in SOD and GPX levels in pancreatic tissue and fundic mucosa, is postulated to be behind the protective effect induced by Omega-3 in this study. These actions help stabilize the reactive radicals, preserve the cellular integrity, and restrain the hazards of EDs on both pancreas and stomach. Moreover, Omega-3 treatment was found to enhance systemic insulin sensitivity [63].

In summary, this study demonstrated that PH induced pancreatic and gastric mucosal injury. Omega-3 can significantly attenuate these effects. Induction of oxidative stress in the tissue is a possible mechanism of ED harmful effect, and the anti-inflammatory and antioxidant activity of Omega-3 could be a possible protective mechanism. Further studies on a larger series would be beneficial in order to better comprehend the mechanisms underlying these phenomena.

## Supporting Information

S1 File SPSS file include the raw data of the study variables. (SAV)
